# Phosphatidic acid inhibits SCAB1-mediated F-actin bundling in *Arabidopsis*

**DOI:** 10.1080/15592324.2022.2092346

**Published:** 2022-06-26

**Authors:** Haiqi Fu, Xinhao Yang, Rong Hao, Xiuli Han, Shu Song, Yan Guo, Yongqing Yang

**Affiliations:** College of Biological Sciences, China Agricultural University, Beijing, Haidian, China

**Keywords:** *Arabidopsis thaliana*, PA, SCAB1, F-actin bundling

## Abstract

Stomatal closure-associated actin-binding protein 1 (SCAB1) regulates stomatal closure by mediating actin filament reorganization in *Arabidopsis thaliana*. Our previous study showed that phosphatidylinositol 3-phosphate (PI3P) binds to SCAB1 and inhibits its oligomerization, thereby inhibiting its activity on F-actin in guard cells during stomatal closure. In this study, we show that another phospholipid, phosphatidic acid (PA), also binds to SCAB1 and inhibits its actin-bundling activity but not its actin-binding activity. F-actin bundling was promoted *in vivo* by treating Col-0 seedlings with *n*-butanol, a suppressor of PA production, but this effect was absent in the *scab1* mutant. These results indicate that the signaling molecule PA is involved in the modulation of SCAB1 activity in F-actin reorganization.

Microfilament-mediated stomatal movement is essential for plants coping with drought stress. SCAB1 (stomatal closure-associated actin-binding protein 1) is a plant-specific actin-binding protein that bundles and stabilizes F-actins to regulate the stability and rearrangement of F-actin in guard cells during ABA-induced stomatal closure.^1^ The SCAB1 protein contains a fused immunoglobulin and pleckstrin homology (Ig-PH) domain, which is an atypical binding site for phosphoinositides.^2^

Membrane phospholipids have been a key focus of plant research in recent years due to their important roles in cellular activities such as membrane rearrangement, cytoskeletal dynamics, and responses to phosphorus deficiency, cold stress, and salt stress.^3–7^ We previously found that phosphatidylinositol 3-phosphate (PI3P) specifically binds to SCAB1 and inhibits its oligomerization, thereby regulating its F-actin-bundling activity in Arabidopsis. This finding suggested that PI3P-mediated regulation of SCAB1 activity may affect F-actin reorganization during ABA-induced stomatal closure.^8^

Phosphatidic acid (PA), a phospholipid derivative that can be produced by the phospholipase D (PLD) pathway or the phospholipase C (PLC)-diacylglycerol kinase (DGK) pathway, was reported to regulate salt stress response in Arabidopsis.^9–11^

In this study, we investigated whether other phospholipids might affect the microfilament regulation function of SCAB1. Using an in vitro protein-lipid overlay assay, we screened many phospholipids for their SCAB1 protein binding activity and found that PA could bind to SCAB1 (Figure 1).

**Figure 1.**
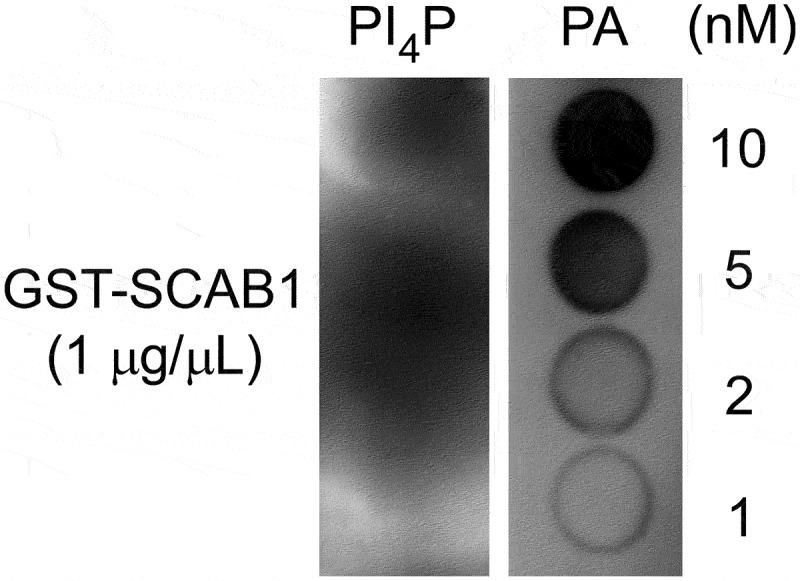
PA binds to SCAB1. A, SCAB1 binds to PA in a protein-lipid overlay assay. Hydrophobic membranes spotted with different concentrations (1, 2, 5, and 10 nM) of PA or PI4P (control) were incubated with 1 μg/mL purified SCAB1 and immunoblotted with anti-GST antibodies. PA was dissolved in methanol.

We previously found that SCAB1 functions as an actin binding protein to regulate the stability of F-actin. SCAB1 bundles actin filaments to promote their stability and regulate the actin reorganization associated with stomatal closure.^1^ Since we also found that PI3P binds to SCAB1 and inhibits its F-actin-stabilizing and -bundling activity in vitro,^8^ we wanted to determine whether PA also affects the bundling activity of SCAB1. To assess the bundling activity of SCAB1, we performed a low-speed co-sedimentation assay. Preassembled F-actin (2 μM) was incubated with SCAB1 (1 μM) and centrifuged at 5,000 *g* for 30 min. The supernatants and pellets were then analyzed by SDS-PAGE (Figure 2a). In the absence of SCAB1, most of the F-actin appeared in the supernatant following low-speed centrifugation, and the amount of F-actin in the pellet fraction increased when the F-actin was pre-incubated with SCAB1. However, when 20 μM PA was added to the F-actin along with SCAB1, the amount of F-actin in the pellets decreased compared with the samples preincubated with SCAB1 alone. These results demonstrate that the ability of SCAB1 to bundle actin filaments was inhibited by PA.

**Figure 2.**
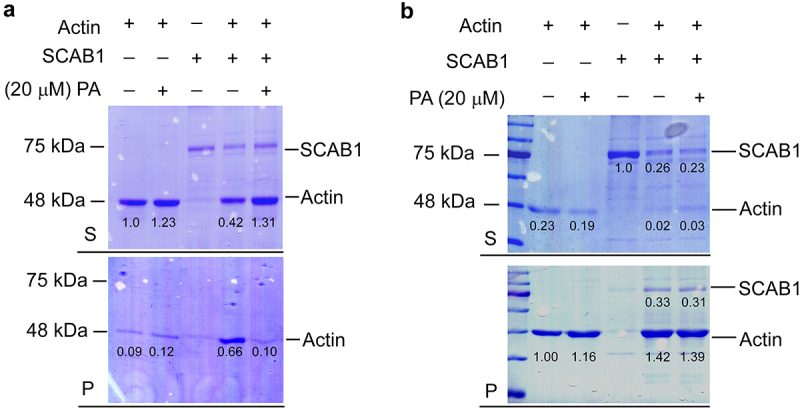
(a) Low-speed co-sedimentation assays of SCAB1 with F-actin. Preassembled F-actin was incubated with SCAB1 (1 μM) treated with or without 20 μM PA. SCAB1 and F-actin were centrifugated at 5,000 *g* for 30 min and analyzed by SDS-PAGE. (b) High-speed co-sedimentation assays of SCAB1 with F-actin. Preassembled F-actin was incubated with SCAB1 (1 μM) treated with or without 20 μM PA. SCAB1 and F-actin were centrifugated at 100,000 *g* for 30 min and analyzed by SDS-PAGE.

SCAB1 has a distinctive protein structure that enables it to bundle F-actin. Its ABD (Actin Binding Domain) is responsible for actin-binding activity, and its CC domain is responsible for the formation of the antiparallel helical hairpins of oligomeric SCAB1^2^. To determine why PA inhibits the bundling activity of SCAB1, we performed a high-speed co-sedimentation assay to assess whether the actin-binding activity of SCAB1 is affected by PA. SCAB1 was incubated with or without preassembled F-actin and then centrifuged at 100,000 *g* for 30 min. The supernatants and pellets were analyzed by SDS-PAGE (Figure 2b). In the absence of F-actin, most of the SCAB1 appeared in the supernatant. The amounts of SCAB1 in the pellets were increased when SCAB1 was incubated with F-actin, indicating that SCAB1 could bind F-actin. The addition of 20 μM PA to the F-actin and SCAB1 before centrifugation had no effect on the amounts of SCAB1 protein in the pellets relative to the results in the absence of PA. This suggested that PA inhibits the actin bundling activity of SCAB1 by some mechanism other than disrupting the binding of SCAB1 to F-actin.

To investigate whether PA could affect microfilament status *in vivo*, we used *n*-butanol as an inhibitor of PA production. *n*-Butanol decreases PA production by PLD by competing with H_2_O as a hydroxyl donor for the phosphatidyl moiety.^12,13^ fABD2, an F-actin binding protein, can be used to observe the status of microfilaments.^1^ Transgenic plants of fABD2-GFP in Col-0 and fABD2-GFP in *scab1* were grown on MS medium for 6 days and then treated in liquid MS medium with or without *n*-butanol (0.4%, v/v) for 24 hours before observing the microfilaments by confocal microscopy. An isomer of *n*-butanol, 2-butanol (0.4%, v/v), was used as a control. Treating the seedlings with 2-butanol had no significant effect on the status of the microfilaments relative to the untreated controls. In contrast, F-actin bundling was increased in Col-0 but not the *scab1* mutant after treatment with *n*-butanol (Figure 3). These results demonstrate that PA inhibits bundling of F-actin in Arabidopsis and that this inhibition is mediated by SCAB1.

**Figure 3.**
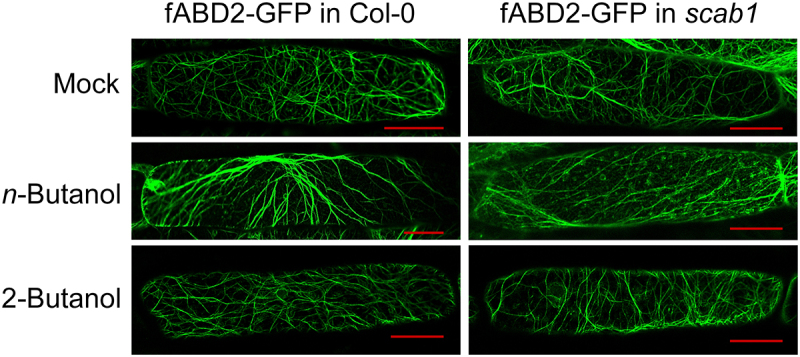
Confocal images of fABD2-GFP-labeled microfilaments (MFs) in hypocotyl epidermal cells in the wild type (Col-0) or scab1 background. Six-day-old transgenic fABD2-GFP/Col-0 and fABD2-GFP/*scab1* seedlings were treated in liquid MS medium with (top) or without (middle) 0.4% (v/v) *n*-butanol for 24 h. MS medium with 0.4% (v/v) 2-butanol (bottom) was used as a control. Scale bars, 25 μm.

In this study, we observed the following: First, PA bound to SCAB1 in an *in vitro* assay; second, PA inhibited SCAB1-mediated F-actin bundling *in vitro* (in a low-speed co-sedimentation assay) and *in vivo* (by the observation of microfilaments in plants); and third, PA did not affect the actin binding activity of SCAB1.

The CC domain of SCAB1 is responsible for the protein adopting antiparallel helical hairpins to form oligomers. PI3P binds to the CC domain of SCAB1 and disrupts the dimerization of SCAB1, thereby inhibiting the F-actin bundling activity of SCAB1^8^. We found that PA inhibited SCAB1-bundling activity without affecting its actin-binding activity. We propose that PA may disrupt the dimerization of SCAB1 to inhibit the bundling activity of SCAB1.

Microfilament dynamics are involved in responses to several stresses such as drought stress^1^ and alkaline stress.^14^ PA levels increase in response to salt stress.^9,10^ Here, we showed that PA can regulate SCAB1 bundling activity, suggesting that SCAB1 or microfilament dynamics may be involved in the salt stress response.

PA has been reported to promote microtubule polymerization and bundling under salt stress by binding to Microtubule-Associated Protein 65–1 (MAP65-1), a regulator of microtubule organization.^15^ During the response to salt stress, plant cells undergo microtubule depolymerization and reorganization. In this study, we showed that PA inhibits SCAB1-mediated F-actin bundling of microfilaments, suggesting that microtubules and microfilaments may play different roles in cytoskeleton-mediated plant cell responses to stress conditions.

## Materials and methods

### Protein-lipid overlay assay

GST-SCAB1 was purified from *E. coli* carrying the recombinant plasmid *pGEX-6p1-SCAB1*. The strips and membranes were prepared by spotting the indicated amounts of PA onto polyvinylidene difluoride membranes. The strips and membranes were allowed to dry for 1 h at room temperature and then incubated with 1 μg/mL purified GST-SCAB1 for 2 h at room temperature in a solution containing 5% skim milk, 20 mM Tris-HCl (pH 8.0), and 150 mM NaCl. The membranes were subjected to five 5-min washes with washing solution (20 mM Tris-HCl, 150 mM NaCl, pH 8.0) and then incubated with anti-GST-SCAB1 antibodies (1:3000 dilution) for 1 h with gentle agitation at room temperature. The membranes were subjected to five 5-min washes with washing solution, and the membranes were incubated with goat anti-rabbit HRP Ab (Bio-Rad, Hercules, CA, USA, 5,213–2,504) (1:5,000 dilution) for 1 h with gentle agitation at room temperature. Finally, the membranes were subjected to five 5-min washes with washing solution and incubated in Clarity western ECL substrate chemiluminescent detection reagent (Bio-Rad) for 3 min prior to image acquisition.^8^

### F-actin co-sedimentation assay

A high-speed co-sedimentation assay was used to determine the effects of PA on the F-actin-binding activity of SCAB1. SCAB1 was dialyzed for 1 h against 1× KMEI buffer (10 mM imidazole, 100 mM KCl, 1 mM MgCl_2_, and 1 mM EGTA, pH 7.0). Protein concentration was determined using the Bio-Rad protein assay kit. Actin was purified from rabbit skeletal muscle acetone powder as described by Pardee and Spudich (1982) in G buffer (5 mM Tris-HCl, pH 8.0, 0.2 mM ATP, 0.1 mM CaCl_2_, 0.5 mM DTT, and 0.01% NaN_3_). SCAB1 (1 μM) was pre-incubated with or without 20 μM PA at 4°C for 30 min. The samples were then incubated with pre-formed F-actin (polymerized from 5 μM G-actin) at 30°C for 30 min and centrifuged at 100,000 *g* for 30 min. Samples from the pellets and supernatants were then analyzed by SDS-PAGE.

For low-speed co-sedimentation assays, 1 μM SCAB1 was incubated with 2.0 μM preassembled F-actin for 60 min at 23°C. After centrifugation at 5,000 *g* for 30 min at 4°C, the supernatants and pellets were separated, subjected to SDS-PAGE, and stained with Coomassie Blue.

### Observation of F-actin status in hypocotyl epidermal cells

*Arabidopsis thaliana* seedlings were grown in a growth chamber at 23°C. F-actin was observed in hypocotyl epidermal cells of 6-d-old transgenic seedlings harboring *Pro35S::fABD2:GFP*.^1^ The seedlings were treated in liquid MS medium with or without *n*-butanol (0.4%, v/v) for 24 hours. MS medium with 0.4% (v/v) 2-butanol (bottom) was used as a control. The actin filaments were observed using an SP8 confocal microscope (Leica).
